# Anti-Aging Potential of Avocado Oil via Its Antioxidant Effects

**DOI:** 10.3390/ph18020246

**Published:** 2025-02-12

**Authors:** Olin Torres-Isidro, Marcela González-Montoya, Manuel Alejandro Vargas-Vargas, Ulises Florian-Rodriguez, Claudia Isabel García-Berumen, Rocío Montoya-Pérez, Alfredo Saavedra-Molina, Elizabeth Calderón-Cortés, Alain Raimundo Rodríguez-Orozco, Christian Cortés-Rojo

**Affiliations:** 1 Instituto de Investigaciones Químico-Biológicas, Universidad Michoacana de San Nicolás de Hidalgo, Morelia 58030, Michoacán, Mexico; olin.torres@umich.mx (O.T.-I.); marceglezmon@gmail.com (M.G.-M.); manuel.alejandro.vargas@umich.mx (M.A.V.-V.); 0620929h@umich.mx (C.I.G.-B.); rocio.montoya@umich.mx (R.M.-P.); francisco.saavedra@umich.mx (A.S.-M.); 2 Facultad de Químico Farmacobiología, Universidad Michoacana de San Nicolás de Hidalgo, Morelia 58240, Michoacán, Mexico; 1900476d@umich.mx; 3 Facultad de Enfermería, Universidad Michoacana de San Nicolás de Hidalgo, Morelia 58260, Michoacán, Mexico; elizabeth.calderon@umich.mx; 4 Facultad de Ciencias Médicas y Biológicas “Dr. Ignacio Chávez”, Universidad Michoacana de San Nicolás de Hidalgo, Morelia 58020, Michoacán, Mexico; alain.rodriguez@umich.mx

**Keywords:** *Persea americana*, aging, lifespan, healthspan, senescence, phytosterols, oleic acid, mitochondria, oxidative stress, inflammation

## Abstract

Aging is a process characterized by tissue degeneration, increased susceptibility to chronic degenerative diseases, infections, and the appearance of neoplasms, which leads to disability and a reduction in the length and quality of life. This phenomenon is the result of the convergence of multiple processes, including mitochondrial dysfunction, fibrosis, inflammation, dysregulation of cell death processes, and immunosenescence. These processes have as their point of convergence an increase in the production of ROS. Avocado oil (*Persea americana* Mill.) contains a diverse array of bioactive compounds, including oleic acid, phytosterols, chlorophylls, xanthones, xanthines, and carotenoids. These bioactive compounds have the capacity to modulate the excessive production of ROS, thereby reducing the progression of age-related diseases and extending lifespan in experimental models of aging. In addition, several studies have demonstrated the efficacy of avocado oil in mitigating age-related diseases, including hypertension; insulin resistance; diabetes; non-alcoholic liver disease; and degenerative processes such as hearing loss, cognitive decline, neurodegeneration, and impaired wound healing. In light of these findings, it is hypothesized that avocado oil is a promising agent capable of promoting healthspan in later stages of life owing to its direct antioxidant actions and the activation of pathways that enhance endogenous antioxidant levels.

## 1. Introduction

Aging is a complex, multifactorial, degenerative process characterized by progressive accumulation of cellular damage; loss of biochemical, physiological, and immunological functions; and eventually, organ and system failure and end of life span [1,2,3]. This process is accompanied by an increased risk of chronic degenerative diseases, weakness, and disability [4]. A hallmark of aging linked to loss of body functions and tissue damage is cellular senescence, characterized by irreversible cell cycle arrest and secretion of proinflammatory molecules, growth and angiogenic factors, and matrix metalloproteases, collectively referred to as the senescent-associated secretory phenotype (SASP) [5].

**Figure 1:**
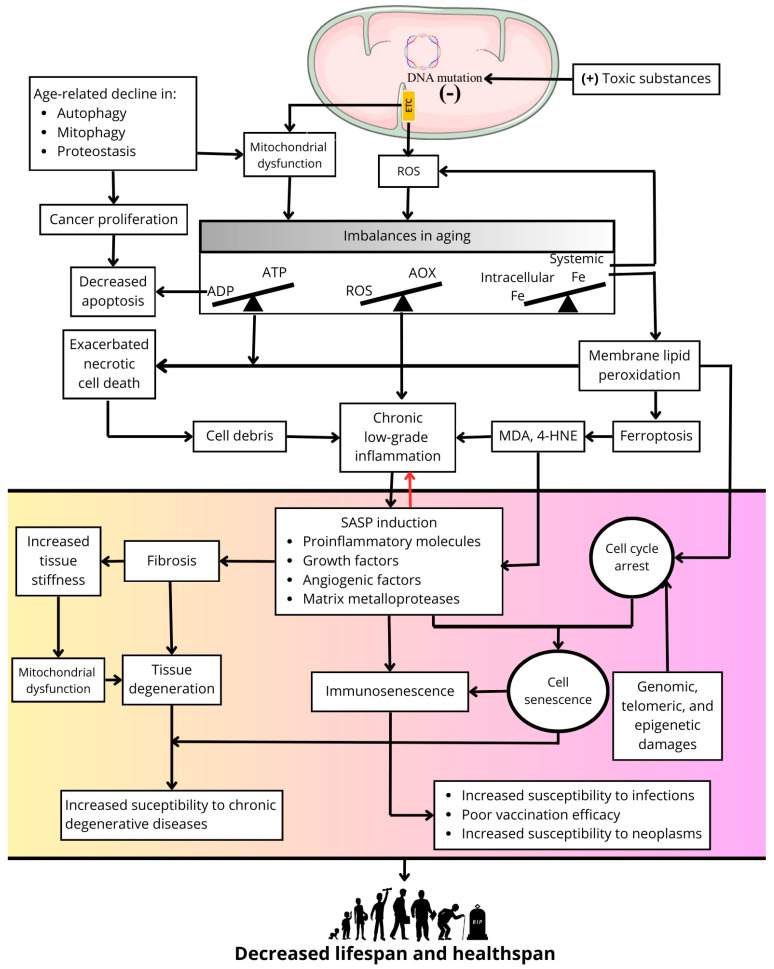
Primary role of ROS in processes related to cellular senescence and aging.

Several theories have been developed to explain the aging process, but none of them alone are completely satisfactory, and in some respects, they complement each other [6]. One of the main theories of aging is the free radical aging theory, developed from the ideas of Rebecca Gerschman in 1954 [7] and Denhan Harman in 1956 [8]. In its simplest conception, this theory proposes that reactive oxygen species (ROS) produced in cells cause damage to macromolecules, leading to accumulation of oxidative damage with age, cellular senescence, and a higher propensity to develop diseases. Despite the accumulating evidence supporting the preponderant role of ROS in aging [9], the validity of this theory has been challenged since caloric restriction, the simplest non-genetic manipulation that universally causes an extension in longevity, results in increased production of ROS in mitochondria [10]. Nevertheless, ROS are a point of convergence in several of the mechanisms that give rise to cellular senescence and body aging, such as mitochondrial dysfunction [11], inflammation [12], fibrosis [13], ferroptosis [14], and senescence [15] (Figure 1). 

With advancing age, mitochondrial DNA accumulates damage due to its higher sensitivity than nuclear DNA to chemical damage because of the high capacity of mitochondria to accumulate positively charged toxic substances due to the negative membrane potential on the matrix side of the mitochondrial inner membrane where the mitochondrial DNA is located [16]. In addition, the proximity of mitochondrial DNA to redox sites of ROS production in the electron transport chain (ETC) favors the accumulation of DNA oxidation products and the appearance of mutations as the organism ages [17]. Proof of these concepts is the excessive accumulation of mitochondrial DNA damage and the appearance of a premature aging phenotype in diseases such as Cockayne Syndrome [18] or xeroderma pigmentosum [19]. The accumulation of mitochondrial DNA damage causes a decline in mitochondrial function, increasing the production of ROS, which together with a decline in antioxidant enzymes with age, produces a redox imbalance resulting in a generalized state of oxidative stress in the cell [20]. In addition, mitochondrial dysfunction also causes an imbalance between the capacity of the cell to synthesize ATP and its ability to respond to ATP demand, which is a feature of aging [21]. Another imbalance characteristic of aging is a systemic decrease in iron content [22] and its excessive intracellular accumulation in tissues [23]. High iron concentrations increase the levels of ROS such as the highly reactive hydroxyl radical. 

Mitochondria, the primary source of ROS in cells, accumulate positively charged (+) toxic substances due to the negative (−) membrane potential in the intermembrane space. This process generates mutations in mitochondrial DNA, dysfunction of the respiratory chain, and oxidative phosphorylation, and causes an increase in ROS production. A decline in quality control processes, such as mitophagy, autophagy, and proteostasis, during the aging process contributes to mitochondrial dysfunction and may promote uncontrolled cell proliferation. Mitochondrial dysfunction and excess mitochondrial ROS generate imbalances between antioxidant availability (AOX) and ROS formation (i.e., oxidative stress) and between energy generation (i.e., ATP synthesis) and energy demand. The latter decreases apoptosis and contributes to the uncontrolled proliferation of cells with defective genetic material. Moreover, an imbalance between circulating iron concentrations and cytosolic iron concentrations results in intracellular iron overload in aging organisms, exacerbating ROS production and contributing to mitochondrial dysfunction and ROS production in the respiratory chain. Increased membrane lipid peroxidation and cell death by ferroptosis is promoted by excess iron. Likewise, oxidative stress and energy deficits promote necrotic cell death. The cellular debris generated by these two types of cell death promotes a state of chronic low-grade inflammation as aging progresses, to which the progressive increase in oxidative stress and the accumulation of lipid peroxidation end products such as 4-hydroxynonenal (4-HNE) and malondialdehyde (MDA) also contribute. Together, these factors induce SASP. Concurrent with cell cycle arrest induced by the accumulation of genomic, epigenetic, and telomeric damage, this results in a cellular senescence phenotype, affecting cells of the immune system and causing immunosenescence and immune deficiency. Furthermore, SASP contributes to alterations in the extracellular matrix, generating fibrosis and increased tissue stiffness. The latter feeds back into mitochondrial dysfunction, and together with fibrosis, promotes tissue degeneration, increasing susceptibility to chronic degenerative diseases in aging. Collectively, these processes contribute to a decline in both lifespan and healthspan.

Concurrently, excess ROS together with an imbalance in mitochondrial energy metabolism leads to uncontrolled necrotic cell death. Additionally, excess iron increases membrane lipid peroxidation and cell death by ferroptosis, the latter being recognized as a form of inflammatory cell death [14]. In contrast, apoptosis is generally decreased during aging, as its execution has a dependency in ATP levels [24,25]. However, apoptosis inhibition is detrimental to tissues homeostasis since, together with an age-related decline in quality control processes such as autophagy, mitophagy, or proteostasis, it allows for the survival of cells with genomic alterations that may favor deleterious processes in aged organisms, such as cancer proliferation [26].

On the other hand, the release of cellular debris resulting from necrotic cell death [27], excess ROS [28], and increased lipid peroxidation [29] may be responsible for stimulating a state of chronic low-grade inflammation present in aging that results in the induction of SASP [30]. This and cell cycle arrest induced by genomic, telomeric, and epigenetic damage or by high levels of lipid peroxidation produce a widespread cellular senescence phenotype in aged organisms [26,31]. In turn, SASP amplifies and perpetuates the chronic inflammation associated with aging at local and systemic levels [32]. In addition, SASP promotes fibrosis processes in organs such as the lungs or kidneys via defective remodeling of the extracellular matrix [33,34], leading to increased tissue stiffness and mechanical damage that feeds back into mitochondrial dysfunction and other defects in cellular function as well as the amplification of fibrosis to neighbor tissues [35]. The sum of all these processes exacerbated during aging results in cellular dysfunction, tissue and organ degeneration, immune system dysfunction (i.e., immunosenescence), increased risk of chronic degenerative diseases, increased susceptibility to infections and neoplasms, and poor vaccination efficacy [19]. This culminates in a decrease in healthspan and in the end of longevity (Figure 1).

Based on the previously described mechanisms of aging that have as a central axis the overproduction of ROS (Figure 1), in the present work, we describe the theoretical basis for proposing avocado oil (AO) as a functional food and a source of molecules that could increase healthspan by delaying the onset of diseases associated with aging due to its antioxidant properties and other biological activities. In the following sections, we describe the origin of avocado, the methods for its extraction, and its commercial importance that justify the feasibility of its use in the general population. We then review the different bioactive components of AO and their modulatory effects on processes involved in the progression of aging. Subsequently, we review some studies on various components of AO that have the potential to improve longevity in experimental models of aging. We also analyze the beneficial effects of AO consumption in experimental models of chronic degenerative diseases related to aging. Finally, we highlight the absence of studies on the potential anti-aging effects of AO and propose a series of targets by which AO could delay the onset of some aging-related diseases with the aim of improving healthspan in aging. 

## 2. The Avocado: Its Origin, Varieties, and Commercial Importance

The avocado is the fruit of the evergreen tree *Persea americana* Mill., which belongs to the family Lauraceae. The avocado is native to tropical America, including the highlands of eastern and central Mexico, Guatemala, Central America, and northern South America (Peru, Ecuador) [36]. It has been spread to almost all tropical and subtropical regions of the world from its natural habitat [37]. The oldest evidence of the presence of avocado in Mesoamerica is from 10,000 years ago in Coaxcatlán, Puebla (Mexico). It is believed that the Aztecs, Mayans, and Olmecs regarded avocados as divine offerings [38]. The name “avocado” is derived from the Nahuatl word “ahuacatl”, signifying “testicle”, an allusion to the fruit’s distinctive shape [39]. Avocado cultivation was already established in Mexico by the time of the Spanish conquest of the Americas [37]. However, avocados did not become a significant commercial crop until the early 20th century. In the early 1950s, avocados began to be accepted as a salad product, and their consumption became more common [40].

The avocado taxonomy encompasses approximately 500 varieties exhibiting diverse morphologies, including sizes, shapes, and colors [41]. However, most of these varieties are not produced on a large scale [42]. The avocado taxonomy is classified into three botanical races: *P. americana* var. Drymifolia (Mexican race), *P. americana* var. Guatemalensis (Guatemalan race), and *P. americana* var. American (Antillean race). The Mexican race avocado skin is very thin with a purple-to-black, dark-green hue and the flesh has an anise-like odor. In contrast, the Guatemalan race exhibits a thicker skin, often with a woody and brittle texture, a rough surface, and a green or black hue. The seeds are generally smaller and tightly packed within the cavity. The Antillean race has a thin to moderately thin skin with a pale green to maroon hue [43]. As for their size, the fruit of the Mexican avocado is small and contains a high percentage of oil, ranging between 16.2/100 g to 32.3 g/100 g, while those of the Antillean are larger and have a lower oil content. On the other hand, the fruit of the Guatemalan avocado has intermediate characteristics between Antillean and Mexican [44]. 

The development of avocado cultivars, which are obtained through human manipulation, involves the selection and cultivation of hybrids between the aforementioned races. An example is the Hass variety, a Guatemalan–Mexican hybrid race, the most widely consumed in the world, which is responsible for 95% of the total commercialized volume in the United States [45]. Other notable varieties include Bacon, Ettinger, Pinkerton, Reed, Fuerte, and Lam [46]. The minimum oil content necessary for marketing avocado fruit is 8%. After maturation, values greater than 20% can occur [47]. In a particular study, 13 varieties of avocado grown in a germplasm bank in Venezuela were analyzed. The study revealed that the oil content in these varieties ranged from 11.23% to 18.80% [48]. Another study analyzed seven avocado varieties at different altitudes in Lebanon. The study revealed that the Fuerte variety had the highest oil content with 21.6%, while the Reed variety had the lowest value, with 9.7% [49].

The global avocado market and trade have exhibited exponential growth over the past two decades, with global production reaching over eight million tons in 2020 [50]. The leading countries contributing to this production are Mexico, the Dominican Republic, Peru, Indonesia, Colombia, and Brazil. Mexico stands as the preeminent avocado-producing nation, accounting for approximately 30% of global output. The majority of avocados traded internationally are destined for the United States and Western Europe [51]. Notwithstanding the commercial significance of avocado, the production volume of AO remains comparatively modest when viewed in contrast to other culinary oils. The primary AO producers are New Zealand, Mexico, Chile, the United States, and South Africa [52]. The market value of AO was estimated at around $430.8 million in 2018 and projected to reach $646 million by 2026 [53]. However, statistics on AO production and commercialization worldwide are very limited [54].

### Extraction Methods for AO

A concise overview of the available methods is provided here, with a more exhaustive review available in the works of Tan [55], Quin and Zhong [56], and Satriana et al. (2019) [57]. Expeller pressing is a mechanical method that uses a screw press arrangement to extract oil from plant materials with high oil content. The avocado pulp is dehydrated due to its high humidity. The yield of AO was found to range from 55.7% to 61.2%. It has been posited that the generation of heat by friction has the potential to adversely affect the quality of the extracted oil [55]. The ultrasonic-assisted aqueous extraction method utilizes cavitation forces produced by acoustic waves, which serve to disrupt the cell walls of cells containing oil. This process gives rise to an emulsion, thereby facilitating the extraction of the oil. This method can be carried out using an ultrasonic bath or an ultrasonic horn transducer [58]. The conventional method of oil extraction involves the use of organic solvents, which exhibit high solubility in various oils, including chloroform, benzene, hexane, acetone, and cyclohexane. During this process, the interactive forces between lipids and the tissue matrix are modified, facilitating oil extraction. However, it should be noted that these solvents can be toxic when consumed. In contrast, the extraction of oil by means of pressing or the utilization of supercritical CO_2_ is more selective and better suited to the food and pharmaceutical industries, as well as to human use and consumption, than the solvent extraction method, despite yielding a lower extraction rate. Other methods, such as cold-pressed, enzyme-assisted centrifugation techniques, or ultrasound-assisted extraction, have also been employed [59].

## 3. Bioactive Compounds in Avocado Oil: Anti-Aging Effects of Its Components Tested Separately

AO is a complex mixture of fatty acids and a minority fraction that contains a wide variety of compounds with antioxidant properties. In this section, we review the mechanisms by which the main fatty acids and antioxidant compounds in AO can improve various harmful processes that give rise to the aging phenotype. This aims to support the hypothesis that AO may possess anti-aging properties. 

### 3.1. Fatty Acids

AO is distinguished by its high proportion of unsaturated fatty acids (UFAs), accounting for approximately 70% of the total fatty acid composition. This percentage is notably higher compared to other commonly consumed edible oils. The fatty acid profile of AO encompasses oleic, palmitic, linoleic, linolenic, and stearic acid. It has been reported that both saturated fatty acids (SFAs) and UFAs activate autophagy through different signaling pathways [60]. Autophagy is a fundamental cellular process that eliminates molecules and subcellular elements via lysosome-mediated degradation to promote homeostasis, differentiation, development, and survival [61]. 

Monounsaturated fatty acids (MUFAs), specifically oleic acid (OAc), are present in high concentrations in AO. The OAc content of AO varies between 47 and 72%, depending on the extraction method and avocado cultivar [62]. OAc has been shown to possess antioxidant and anti-inflammatory properties, which are processes implicated in the aging process [63]. OAc has been shown to directly regulate the synthesis and activities of antioxidant enzymes, and its anti-inflammatory effect may be related to its ability to inhibit proinflammatory cytokines and stimulate anti-inflammatory ones. In addition, AOc has been identified as a natural activator of sirtuin 1 (SIRT1) [64]. It has been reported that SIRT1 activation has neuroprotective effects against age-associated neurological disorders, such as Alzheimer’s disease (AD), and is a therapeutic target for the optimal balance between anti-aging and anti-cancer activities [65,66,67]. OAc demonstrated a substantial reduction in the 25–35 fragment of the beta amyloid peptide (Aβ25–35) mediated toxicity by enhancing cell viability through the mitigation of ROS [68]. In particular, the anti-inflammatory effects of OAc have been linked to OAc-derived oleoyl ethanolamide (OEA), a naturally synthesized compound in the human body whose levels are influenced by dietary oleic acid intake [69]. OEA is an endogenous ligand of the peroxisome proliferator-activated receptor alpha (PPAR-α), a nuclear receptor protein that acts as a transcription factor. It has been suggested as a potential therapeutic agent for the treatment of obesity and has also been shown to improve insulin sensitivity and produce selected anti-inflammatory effects as well as antioxidant activity [64,70]. However, contradictory findings have emerged from studies demonstrating that OA elevates mitochondrial ROS production and oxidative damage, along with a decline in endothelial nitric oxide synthase activity, in murine dermal fibroblasts and endothelial cells following brief exposure [71,72]. In addition, OAc has been demonstrated to suppress iron-overload-induced damage in cultured mammalian cell lines by preventing ferroptosis. This protective effect was observed to be diminished in animals lacking the nuclear hormone receptor NHR-49, which shares functional homology with the mammalian PPAR-α. Furthermore, OAc was observed to inhibit liver lipid peroxidation and reduce liver damage in a mouse model of iron overload [73].

The second reported component of the AO fatty acids is palmitic acid (PAc), which is a saturated fatty acid (SFA) and can account for up to 30% of the total fatty acids, depending on the avocado variety, altitude, and extraction conditions [62]. However, excessive PAc consumption has been associated with adverse effects. PAc has been shown to promote inflammatory pathways through the production of ROS and the overproduction of the pro-inflammatory molecule IL-6 [74]. Additionally, elevated levels of PAc in the blood have been observed in obese patients, and these levels have been associated with inflammatory responses via toll-like receptor (TLR)2 and 4 [75]. Furthermore, PAc can be converted into phospholipids, diacylglycerol, and ceramides, which can in turn induce insulin resistance [76]. Conversely, a study demonstrated that PAc could extend lifespan and enhance stress resistance in the nematode model, which has potential applications in health promotion, including the delay of aging and the reduction of risk for age-related diseases [77].

AO is a significant source of polyunsaturated fatty acids (PUFAs), including linoleic and linolenic acids. Linoleic acid, an omega-6 PUFA, ranges from 6.6% to 20.8% in AO, depending on the avocado variety, altitude, and extraction conditions [62]. However, a study reported 48.77% linoleic acid in Hass AO extracted by the Soxhlet method [58]. The potential of PUFAs to modulate inflammatory responses in the central nervous system, particularly in microglia, which are the primary mediators of inflammation in age-related conditions such as AD, has been demonstrated [78]. In particular, α-Linolenic acid (ALA), an omega-3 PUFA, is also present in AO in ranges from 0.1% to 2.6%, depending on the avocado variety, altitude, and extraction conditions [62]. Some studies have found that omega-3 PUFAs might have the potential to improve sarcopenia by repairing mitochondrial function by promoting mitophagy and fighting oxidative stress [79]. In turn, ALA has demonstrated its role as an anti-inflammatory and antioxidant agent within AO. Doses of 60 mg/kg administered orally for a period of six weeks exhibited particularly notable anti-inflammatory and antioxidant potential against cadmium-induced oxidative stress, neuroinflammation, and neuronal apoptosis in the adult mouse brain, accompanied by a reduced ROS production, and it has also been demonstrated to enhance the expression of heme oxygenase-1 (HO-1) and nuclear factor-2 erythroid-2 (NRF2), a transcription factor that plays a pivotal role in regulating cellular redox homeostasis. It is notable that the decline in NRF2 levels with age contributes to the aging phenotype. Additionally, the decline in NRF2 with age has been associated with a decrease in NRF2 signaling, including loss of proteostasis, genomic instability, telomere attrition, and epigenetic alterations [80]. Furthermore, ALA has been associated with enhanced insulin and insulin-like growth factor 1 (IGF) release in human astrocytes, thereby fostering mitochondrial biogenesis and maintaining mitochondrial dynamics [81]. 

### 3.2. Phytosterols

Phytosterols are triterpenoid compounds that are found in plants and have a chemical structure similar to that of cholesterol. These compounds compete with cholesterol for intestinal absorption. The phytosterol content of AO is approximately 4.5 mg/g [82], with β-sitosterol comprising approximately 75–80% of this [83]. Research has indicated that β-sitosterol is the predominant phytosterol in AO and has demonstrated protective effects against inflammation, diabetes, and AD by inhibiting IL-6, oxidative stress, cyclooxygenase, and nitric oxide [84]. This is of particular relevance given the heightened inflammatory responses and age-related diseases that accompany the aging process. In addition, a study reported that a 40 mg/kg dosage of β-sitosterol could mitigate the deleterious effects of gamma irradiation in arthritic rats, suggesting its potential to effectively counteract inflammation and oxidative stress by suppressing MDA production and COX-2, inducible nitric oxide synthase. The anti-inflammatory and antioxidant properties of β-sitosterol have been demonstrated in a study that examined its effects on articular and extra-articular organs, including the liver, heart, and kidneys. The study found that the treatment led to a decrease in MDA production and the expression of iNOS, COX-2, myeloperoxidase (MPO), and nuclear factor kappa-β (NF-κB) in these organs. In addition, treatment with β-sitosterol offered protection against oxidative damage from CuSO_4_ in biological models of inflammation (zebra fish and macrophages RAW 264.7) by upregulating the expressions of superoxide dismutase (SOD) and glutathione peroxidase (GPx) [85,86]. Furthermore, β-sitosterol has been shown to regulate serum lipid levels and enhance immune function, antioxidant status, and intestinal morphology in broilers, leading to a reduction in jejunal tumor necrosis factor alpha (TNF-α) and ileal interleukin 1 beta (IL-1β), as well as mRNA relative expression levels of jejunal TLR4 and ileal MyD88, whereas it increased contents of jejunal immunoglobulin G (IgG), ileal secreted IgA and glutathione, jejunal catalase (CAT) activity, and NRF2 mRNA relative expression levels, improving intestinal antioxidant status [87].

### 3.3. Tocopherols

AO is abundant in tocopherols, predominantly alpha (α) and gamma (γ), which are highly bioactive antioxidants [88]. α-tocopherol plays a crucial role in neutralizing free radicals, stabilizing the UFA content, preventing the oxidation of fats and phytosterols, preserving oil quality, and extending the shelf life of fats and other food products [62]. γ-tocopherol, a primary form of vitamin E, possesses significant lipophilic antioxidant properties. Notably, γ-tocopherol possesses the unique ability to neutralize reactive nitrogen species by forming 5-nitro-γ-tocopherol, thereby providing superior protection for mitochondrial function [89]. A study reported that α- and γ-tocopherols protected murine microglial BV-2 cells, triggering an improvement in mitochondrial and peroxisomal dysfunction involved in neurodegeneration caused by 7-ketocholesterol (7-KC), which is increased in the body fluids and tissues of patients with neurodegenerative diseases [90]. Furthermore, α-tocopherols, including α-tocopherol and α-tocopherol hydroquinone (a derivative of vitamin E), have been observed to enhance the phosphorylation levels of PI3K, AKT, and mTOR in rat bone marrow-derived mesenchymal stem cells (BMSCs) in response to H_2_O_2_ stimulation. In addition, these compounds have been shown to suppress ferroptosis [91,92,93]. A study demonstrated that γ-tocopherol exhibited superior efficacy in enhancing mitochondrial functions when compared with α-tocopherol. This enhancement was achieved through the regulation of mitochondrial membrane permeability, which was achieved by affecting the mRNA expression of *VDAC1* and *CypD*. The study further revealed that γ-tocopherol prevented apoptosis by reducing cytochrome *c* release and the BAX/Bcl-2 ratio. Additionally, it functioned as a potent antioxidant, thereby preventing the accumulation of beta-amyloid peptide (Aβ) in an in vitro model of Alzheimer’s disease [94].

### 3.4. Carotenoids

Carotenoids are a class of terpenoid pigments, and they are responsible for colors ranging from yellow to orange-red. The total reported in AO ranges between 1 and 3 mg/kg in cold-pressed AO and up to 28 mg in AO derived from seven varieties of avocados [62,95]. Furthermore, 9.7–20 mg/kg extracted at different temperatures and reaction conditions by ultrasonic and microwave treatment of avocado pulp and oil separation by centrifugation [95,96] have also been reported. These pigments can be categorized into two distinct groups: carotenes, which are hydrocarbons devoid of oxygen, and xanthophylls, which contain oxygen in their structure. A distinguishing feature of carotenes and xanthophylls is their structural and functional diversity [97]. They fulfill a pivotal role as antioxidants by scavenging and neutralizing free radicals, thereby safeguarding cells from oxidative damage [98]. Carotenoids have been shown to protect cells, tissues, and organs from the damaging action of singlet oxygen by scavenging free radicals via transferring electrons, creating adducts, and moving hydrogen atoms [99]. Furthermore, carotenoids have been demonstrated to protect cells from lipid peroxides and oxygen radicals. Research on the human inner mitochondrial membrane enzyme β-carotene oxygenase 2 (BCO2) has led to the development of mitochondrial-targeted carotenoids. BCO2 is involved in the catalytic activities of both provitamin and non-provitamin A carotenoids, which results in the formation of apo-carotenoids. These carotenoids have a role to play in mitochondrial oxidative stress. Low-grade inflammation and metabolic dysfunction are also associated with BCO2 deficiency, leading to excessive superoxide production in mitochondria. This, in turn, results in the suppression of mitochondrial superoxide dismutase levels and the impairment of mitochondrial respiratory complexes [100]. The most abundant carotenoids in AO are lutein, neoxanthin, zeaxantin, violaxanthin, and β-carotene, and their content is dependent on the extraction methods and temperature conditions of the saponification method [62,101]. Lutein’s impact on aging is multifaceted, involving the downregulation of the NF-κB pathway and NOD-like receptor thermal protein domain-associated protein 3 (NLRP3) inflammasome as well as the upregulation of antioxidant enzymes such as SOD and catalase. These processes contribute to reducing oxidative stress and inflammation associated with aging [102]. Moreover, lutein supplementation has been shown to enhance neurogenesis and neuroplasticity, thereby linking its neuroprotective effects with its antioxidant and immunomodulatory activities through key signaling pathways mediated through the regulation of NRF2/ERK/AKT, NIK/IKK, PI3K/AKT, MAPK/ERK, and JNK. In addition, lutein supplementation has been demonstrated to prevent neuronal dysfunction by resolving mitochondrial abnormalities, excitotoxicity, and metal accumulation in the central nervous system [103]. 

The antiaging effects of β-carotene were demonstrated through the regulation of the KAT7-P15 signaling axis. The KAT7-P15 axis refers to a signaling pathway where the histone acetyltransferase KAT7 modulates the expression or activity of P15, influencing key biological processes such as cell aging, inflammation, and oxidative stress [102]. Furthermore, a study demonstrated the cytoprotective potential of β-carotene, lutein, and neoxanthin by measuring the cytotoxic effect of H_2_O_2_ and revealing that neoxanthin pretreatment exhibited superior protection compared to β-carotene and lutein against cell death induced by H_2_O_2_ and protected mitochondrial membrane potential [104]. Additionally, pretreatment for 3 hours with neoxanthin, a xanthophyll pigment, exhibited anticancer, antioxidant, and chemopreventive activity in colon and hepatic cancer cells by reducing oxidative stress and causing mitochondrial dysfunction and apoptosis induction by increasing Bax expression and preventing Bcl-2 expression [105,106].

### 3.5. Xanthones

Although AO has not been identified as a significant source of these compounds, recent research in our laboratory has revealed the presence of a xanthone, known as Yahxanthone [107], within an unsaponifiable AO fraction. This xanthone exhibits a distinctive chemical structure and has been associated with the previously reported cellular toxicity in murine leukemia cells [108]. This unexplored xanthone represents a potential subject for study due to its limited information and structural potency. Xanthones are polyphenolic compounds characterized by a structure consisting of a benzopyranone ring and containing an oxygen atom in the heterocyclic ring. Xanthones are a significant class of oxygenated heterocycles, and their role in medicinal chemistry is well established [109]. The biological activities exhibited by this class of compounds are associated with their tricyclic scaffold, yet they demonstrate variability depending on the nature and/or position of the different substituents [109]. Notably, xanthones have demonstrated antitumor activity by targeting PKC modulation [110]. Furthermore, it has been documented that natural and synthetic xanthone derivatives counteract oxidative stress via NRF2 modulation in stimulated human macrophages [111].

### 3.6. Chlorophylls

As demonstrated in [112], AO exhibits high concentrations of chlorophyll (40–60 µg/g) in cold-pressed AO. A study identified four principal compounds in AO, including pheophytin *a* (1.1 µg/g), pheophytin *b* (2.2 µg/g), chlorophyll *a* (4.9 µg/g), and chlorophyll *b* (5.1 µg/g) [62]. Pheophytin *a* and *b* are the most prevalent chlorophylls, and they have been documented as antioxidant and anticancer agents [113]. Regarding metabolism, they are transformed into their corresponding pheophorbides and pheophytins, and they are absorbed at similar intestinal rates as carotenoids [114]. However, they also act as prooxidant compounds in lipophilic environments under light conditions [114]. Pheophytin *a* and hydroxy pheophytin have been documented as potent inducers of oxidoreductase-1 (NQO-1) in liver cells, thereby inducing liver detoxification and enhancing the redox state [115]. Chlorophyll *a* and pheophytin *a* have been shown to possess significant anti-inflammatory activity in animal models, including Swiss albino mice and Wistar rats. These compounds have been observed to inhibit TNF-α gene expression induced by LPS, and both chlorophyll *a* and chlorophyll *b* have demonstrated the capacity to suppress NF-κB [116]. In addition, pheophytin *a* has been reported to inhibit cellular migration, induce DNA fragmentation, arrest the cell cycle at the S phase, and reduce mitochondrial membrane potential. Specifically, chlorophyll *a* significantly reduced edema in mice and rats and also in HEK293 cells [116] and was found to induce apoptosis [117]. Furthermore, chlorophyll *a* was found to inhibit TNF-α gene expression induced by LPS, and both chlorophyll *a* and chlorophyll *b* demonstrated the capacity to suppress NF-κB [116]. In addition, pheophytin a has been reported to inhibit cellular migration, induce DNA fragmentation, arrest the cell cycle at the S phase, reduce mitochondrial membrane potential, and promote apoptosis [117].

### 3.7. Phenolic Compounds

The content of total phenol compounds in AO exhibits significant variability, with reported values reaching up to 408 mg/L [62]. However, the presence of phenolic compounds is found to be more pronounced in avocado peels compared to the pulp. Consequently, the utilization of avocado peels during oil extraction may potentially augment the concentration of these compounds, as evidenced by the observation that the highest phenolic content was attained when oil was extracted from unripe and unpeeled avocados [118]. 

AO has been found to contain a variety of phenolic substances, including phenolic acids (hydroxycinnamic acids or hydroxybenzoic acids), such as vanillic acid, chlorogenic acid, p-vanillin, ferulic acid, and tyrosol, quercetin, apigenin, and luteolin in the 7-glucoside form, as well as flavonoids and procyanidins, which have been reported in avocado extracts [119]. Gallic acid, p-coumaric acid, ferulic acid, and quercetin were identified in avocado pulp oil, exhibiting variations in their concentrations based on factors such as ripening and peeling, and demonstrating antioxidant properties [120]. Phenolic acids have been observed to enhance lifespan, heat-stress resistance, and chemotaxis at micromolar concentrations. Furthermore, the influence of aged phenolic compounds on mitochondrial membrane potential has been demonstrated, with vanillic acid exhibiting particular efficacy in this regard [121]. Quercetin, a prominent polyphenol, possesses antioxidant and anti-inflammatory properties capable of impeding the synthesis of inflammatory cytokines and enzymes. This renders it a promising therapeutic agent for various inflammatory conditions [122]. The neuroprotective effects of quercetin have been examined in human SH-SY5Y neuronal cells subjected to H_2_O_2_-induced apoptosis. This process has been shown to suppress induced toxicity and lactate dehydrogenase release. Additionally, it has been observed to downregulate the pro-apoptotic Bax gene and upregulate the anti-apoptotic Bcl-2 gene [123]. As illustrated in Table 1, the paper presents a compendium of the primary compounds that are present within the oil, along with their respective mechanisms of action, which collectively contribute to the observed anti-aging effect.

## 4. Potential of Individual Components of AO to Extend Lifespan

As outlined in the previous section, the various components of AO exhibit individually distinct biological activities that have the potential to mitigate or delay the onset of an aging phenotype. Given the correlation between reduced aging and increased longevity, the subsequent section reviews experimental evidence on the impact of the individual compounds of AO on longevity in various aging models. This provides further evidence to support the idea that avocado oil may be used as an anti-aging agent.

### 4.1. Effect of Fatty Acids on Longevity Experimental Models

Research has demonstrated that OAc enhances the activity of SKN-1A/NRF1, an ER-resident transcription factor, in *C. elegans*, thereby increasing their resistance to oxidative stress. This activation is facilitated by enhancing the efficiency of ER-associated degradation in a lipid droplet-dependent manner, independent of proteasome activity. SKN-1A, in turn, has been shown to reduce steatosis by altering the lipid metabolism transcriptome and to promote longevity, either through endogenous accumulation, dietary supplementation, or by affecting histone methylation [136]. As is well documented, reproduction in *C. elegans* is an energetically costly process, leading to significant depletion of energy reserves, particularly fat, which are crucial for maintenance and survival. Extensive research has demonstrated a clear correlation between increased reproductive activity and a decreased lifespan. However, treatment with OAc mitigates this fat loss by upregulating FAT-7, a desaturase enzyme key to MUFA biosynthesis. Remarkably, this intervention extends mating-induced life expectancy without compromising reproductive output [137]. 

OAc administration in *P. anserina* extends longevity by reducing ROS through altered mitochondrial respiration, by changing the dynamics of mitochondrial respiration, and by avoiding the use of complexes I and II. This process attenuates ROS production and enhances membrane trafficking. This supports the function of PaAtg24, the ortholog of SNX4 in other organisms, which is crucial for the recycling of PaSNC1. This prevents endosomal accumulation and ROS elevation in ΔPaAtg24 mutants, thereby increasing lifespan [138].

PAc is one of the most predominant fatty acids along with OAc [139]. Research has shown that PAc has the ability to extend the lifespan of *C. elegans* and enhance resistance to oxidative stress. A study demonstrated that these beneficial effects were mediated through the activation of the transcription factor SKN-1/NRF2 signaling pathways [77]. 

ALA has been shown in research studies to enhance the lifespan of *C. elegans*. In a study, treatment with ALA in wild-type worms led to an increase in life expectancy by activating the transcription factors NHR-49/PPARα and SKN-1/NRF2, which function independently of each other. ALA activates NHR-49, which in turn activates the expression of genes involved in beta-oxidation, a process that generates energy from fatty acids through the tricarboxylic acid cycle and mitochondrial respiration. In contrast, SKN-1 is activated by ALA independently of NHR-49, and ALA activates SKN-1 through a separate process from the one that activates NHR-49. Exposure of ALA to air causes oxidation of ALA to a group of compounds called oxylipins, such as 9(S)-HpOTrE. These molecules trigger biological functions, including activation of SKN-1, which leads to increased longevity by activating genes involved in the antioxidant defense response [140].

The data indicate that the fatty acids present in AO have anti-aging effects and increase life expectancy through several mechanisms. These mechanisms include the activation of transcription factors related to antioxidant defense and the promotion of the activation of enzymes such as FAT-7, which promotes the synthesis of MUFA. 

### 4.2. Impact of Carotenoids on Lifespan 

The potential of β-carotene as an anti-aging agent has been demonstrated both in vitro and in vivo. For instance, in a study on H_2_O_2_-induced aging of mesenchymal stem cells, β-carotene was found to reduce the expression of aging markers p16 and p21. As mentioned before, β-carotene mitigated aging by modulating the KAT7-P15 signaling pathway, improving the aging condition of various tissues and organs [102]. 

In addition to β-carotene, other carotenoids, such as canthaxanthin and astaxanthin, have demonstrated the potential to extend the lifespan of *C. elegans* by stimulating antioxidant enzymes, such as SOD and CAT, and reducing markers of oxidative stress, such as MDA [141]. 

Lutein, another carotenoid found in AO, has shown promise in extending the lifespan of *D. melanogaster* by enhancing the activity of intrinsic antioxidant enzymes, thereby reducing oxidative damage and significantly boosting survival rates under conditions of oxidative stress [142]. 

### 4.3. Effect of Chlorophylls on Antioxidant Defense and Longevity 

Chlorophyll has been shown to enhance oxidative stress tolerance and extend the lifespan of *C. elegans* by up to 25%. This increased longevity can be attributed to the activation of the DAF-16/FOXO pathway, a regulatory pathway that controls responses to stress and aging by influencing gene expression related to antioxidant defenses and longevity in various organisms, including humans [143].

### 4.4. Influence of Phytosterols on Aging and Survival 

AO is rich in phytosterols, which have been shown to offer significant health benefits beyond their cholesterol-regulating properties [144]. Research has shown that phytosterols extracted from ginseng can extend the lifespan and promote healthy aging in *D. melanogaster*. Specifically, stigmasterol has been found to activate steroid signaling pathways, suggesting a role in mimicking the effects of steroid hormones, which are known to decline with age. This finding is particularly intriguing for AO, which is rich in phytosterols like β-sitosterol, potentially offering similar benefits in extending healthspan and lifespan through dietary intake [145]. 

### 4.5. Effect of Phenolic Compounds on Lifespan 

The phenolic compounds, particularly protocatechuic, gallic, and vanillic acid, which are present in high concentrations within the unsaponifiable fraction of AO, are known for their antioxidant properties [146]. A study using the *C. elegans* strain PX627, a model for aging, demonstrated that these phenolic acids significantly extend lifespan and improve health parameters such as heat stress resistance and chemotaxis. Notably, while the mitochondrial membrane potential remained unaltered with age, vanillic acid specifically reduced ATP levels in older nematodes, indicating a modulation in energy metabolism. These acids also activated longevity-related pathways, with vanillic acid notably increasing glycolytic activity. The study suggested that the beneficial effects of these phenolic acids on aging involve the activation of the sirtuin pathway, which are proteins implicated in regulating cellular processes like metabolism, DNA repair, and aging, rather than directly altering mitochondrial function [121].

Finally, an extract from *L. octovalvis*, which is rich in flavonoids, polyphenols, and notably β-sitosterol, has shown potential in extending the lifespan of *D. melanogaster*. The effectiveness of this extract appears to be achieved through mechanisms similar to those of dietary restriction and the activation of AMPK in the body fat of adult flies. Notably, β-sitosterol, a prominent constituent of this extract, is also present in substantial amounts in the unsaponifiable fraction of AO. While the study did not isolate β-sitosterol specifically, its presence alongside other bioactive compounds in *L. octovalvis* suggests a molecular basis for anti-aging effects, potentially applicable to compounds found in AO or related substances [147].

The presence of a diverse array of bioactive compounds in AO, including OAc, carotenoids, chlorophyll, phenolic compounds, and phytosterols, suggests the potential for these constituents to exhibit anti-aging effects similar to those observed in the discussed studies. These compounds have been demonstrated to influence pathways associated with longevity, stress resistance, and metabolic regulation across various model organisms, suggesting potential health benefits from AO consumption. The paper presents a compendium of AO compounds for anti-aging and age-related diseases, as shown in Table 2.

## 5. Potential Beneficial Effects of Individual Components of AO on Aging-Related Diseases

As shown in the preceding sections of this work, the individual compounds present in AO have the potential to promote longevity by attenuating the onset of degenerative processes that contribute to the progression of aging. In order to establish a correlation between these effects and a reduced development of age-related diseases [148,149,150] the following section analyzes the effect of different compounds in avocado oil on the development of these diseases. 

### 5.1. Neurodegenerative Diseases

Neurodegenerative diseases, including AD, Parkinson’s disease, Huntington’s disease, and amyotrophic lateral sclerosis, are characterized by a progressive degeneration and loss of functional neurons [150]. Research findings have indicated that the consumption of MUFA is associated with enhanced cognitive function in a Japanese elderly population, as evidenced by a cohort study involving 154 individuals, thereby suggesting a potential beneficial effect against cognitive decline [124]. It has been established that in AD, microglia can become overactivated, resulting in elevated secretion of cytotoxins and inflammatory mediators such as nitric oxide. However, when microglial cells are exposed to PUFA, they exhibit reduced nitric oxide secretion and lower levels of iNOS [78]. In addition, the presence of Aβ damage has been identified as a significant contributing factor to the development of AD, and its modulation has emerged as a promising approach for controlling the onset of AD [68]. Supplementation with OAc has shown promise in the amelioration of amyloidosis in AD models [125]. It is noteworthy that research has indicated that AD-affected brains exhibit significantly diminished concentrations of essential components of brain myelin membranes, including linoleic acid, linolenic acid, and OAc [151,152]. These brains also display reduced levels of xanthophylls, carotenes, and tocopherols [153]. Furthermore, an association has been observed between higher levels of α- and γ-tocopherols in the brain and reduced microglia density, particularly in cortical regions, but not in subcortical brain regions. This association was identified in a study involving 113 participants. The finding suggests that the relationship between tocopherols and AD may be partly explained by the anti-inflammatory effects of tocopherols on microglial activation [131]. A body of research has indicated a positive correlation between β-carotene and enhanced telomerase activity in subjects diagnosed with AD, irrespective of gender. This finding underscores the potential involvement of telomeres in the process of neurodegeneration and neurodegenerative disorders, including AD [134]. Moreover, the administration of lutein at a dosage of 20 mg/day has been associated with an augmentation in carotenoid levels in individuals diagnosed with relapsing-remitting multiple sclerosis. This has led to the observation of promising cognitive benefits, particularly in attention and memory domains [134].

### 5.2. Cardiovascular Diseases 

Cardiovascular diseases such as atherosclerosis, hypertension, and myocardial infarction involve pathological alterations in cardiac and vascular tissues, resulting in hypertrophy, compromised left ventricular (LV) diastolic function, diminished LV systolic capacity, increased arterial stiffness, and impaired endothelial function [154]. Carotenoids have been identified as bioactive compounds with significant implications for stroke prevention [155]. Furthermore, carotenoids have been shown to offer substantial protection against the progression of early atherosclerosis in both human and animal subjects by reducing levels of very-low-density lipoprotein (VLDL) and intermediate-density lipoprotein while concurrently mitigating inflammation and oxidative stress within arterial walls [54]. In a study conducted in vivo, lutein was shown to possess significant antioxidant and anti-inflammatory activity in the aortic tissues of guinea pigs with atherosclerosis [156].

### 5.3. Metabolic Disorders

The presence of chronic hyperglycemia, a hallmark of both type 1 (T1DM) and type 2 diabetes mellitus (T2DM), is associated with macrovascular and microvascular complications [157]. It has been documented that diets enriched with MUFA from OAc sources such as avocado and olive oil in individuals with T2DM exhibit a hypoglycemic effect [158,159]. Furthermore, a prediabetic model was utilized to assess the effects of POA and OAc supplementation at a dose of 100 mg/kg, revealing that POA significantly enhanced insulin sensitivity, reduced glucose levels, and diminished proinflammatory cytokines in adipose tissue, while OAc exhibited modest effects on insulin sensitivity and predominantly decreased inflammatory arachidonic acid metabolites [126]. Research has elucidated the role of β-sitosterol as a crucial component in the reduction of serum lipid levels [129]. Investigations into the potential synergistic effects between OA bioactive compounds and pharmaceutical agents have yielded promising results. Initial studies suggest that the combination of insulin sensitizers, such as thiazolidinediones, with antioxidants like vitamin E may lead to improved outcomes in the treatment of nonalcoholic steatohepatitis [160]. Carotenoids, which have been identified as bioactive compounds with significant implications for ocular health, are constituents of the macular pigment. Carotenoids, in conjunction with n-3 fatty acids, have demonstrated protective effects against macular degeneration through antiangiogenic, anti-inflammatory, and antioxidant mechanisms [155]. Diabetic retinopathy, the most common retinal vascular disease, underscores the importance of these protective properties [161]. Chlorophylls have demonstrated anti-obesogenic properties [162], as evidenced by a study that found that chlorophyll supplementation in early life can effectively retard body weight gain, improve glucose tolerance, as well as reduce low-grade inflammation using a 4-week-old C57BL/6J male mice model of obesity induced by a high fat diet (HFD) [163].

### 5.4. Cancer

Cancer, due to its invasive nature, disrupts tissue integrity, promotes chronic inflammation, suppresses immune surveillance, and progressively depletes systemic resources, thereby exacerbating overall health deterioration [164]. OAc consumption has also been associated with a reduction in cancer risk development (primarily breast, colorectal, and prostate cancer) [165,166]. Moreover, β-sitosterol has exhibited cytotoxic properties when tested against hepatoma cell lines [130]. Carotenoids have been identified as bioactive compounds with significant implications for the treatment of various cancers [167]. The antimutagenic, antigenotoxic, and anticancer properties of chlorophylls further highlight their potential as bioactive compounds deserving thorough investigation [162]. 

### 5.5. Musculoskeletal Disease

Distinct pathological characteristics are exhibited by musculoskeletal diseases, including osteoarthritis (OSA) and osteoporosis. Cartilage degradation is a hallmark of OSA, while reduced bone mineral density is a hallmark of osteoporosis [168]. The incorporation of dietary supplements, such as avocado soybean unsaponifiable (ASU), has been demonstrated to modify the symptoms associated with OSA. The primary constituents of ASU include phytosterols, specifically β-sitosterol, campesterol, and stigmasterol. In addition to these compounds, ASU contains a variety of fat-soluble vitamins, sterols, and triterpenes, as well alcohols and possibly furan fatty acids. These molecules rapidly incorporate into cells and contribute to biological activity in articular chondrocytes. These molecules stimulate the synthesis of collagen and aggrecan by inhibiting inflammatory cytokines such as IL-1, IL-6, IL-8, TNF, and PGE2 through modulation of NF-κB [169].

## 6. Preclinical Studies on AO and Aging-Related Diseases 

Despite the information in the previous sections about the potential of the individual components of avocado oil to extend longevity and mitigate the harmful processes responsible for the progression of aging and its related diseases, there is a lack of research on the direct effect of avocado oil on the aforementioned processes or on longevity. However, our research group and other groups have demonstrated a beneficial effect of avocado oil on some of the most prevalent diseases in aging and in some processes that accompany aging such as hearing loss, cognitive decline, neurodegeneration, and impaired wound healing, which are briefly reviewed below.

Aging has been shown to have multifaceted impacts on bodily functions, increasing disease risks through alterations in gene expression, metabolism, heightened oxidative stress, and antioxidant system imbalances, leading to cellular damage and mortality [170]. A significant aspect of numerous age-related pathologies, including AD, diabetes, kidney disease, hypertension, NAFLD, cancer, and hearing loss, is oxidative stress, which is closely associated with mitochondrial dysfunction [171,172]. This process is further exacerbated by a chronic inflammatory condition known as inflammation aging, which disrupts homeostasis, fosters neurodegeneration, and affects energy balance [171,173].

### 6.1. Antioxidant Effect of AO 

The antioxidant effects of AO have been demonstrated on monkey kidney epithelial cells exposed to rotenone, a known inducer of oxidative stress. AO significantly increased cell viability and reduced markers of oxidative stress such as ROS and lipid peroxidation when co-administered with rotenone. This suggests that AO has significant antioxidant potential and may offer protection against oxidative stress-related damage in various age-related diseases [174]. 

### 6.2. AO in Diabetes 

Streptozotocin-induced type 1 diabetes mellitus (T1DM) in rats significantly increased the content of docosahexaenoic acid (C22:6) in liver mitochondrial membranes, leading to higher levels of lipid peroxidation. This condition impaired mitochondrial respiration, reduced complex I activity, and increased ROS generation with complex I substrates, along with a more oxidized glutathione state, signaling oxidative stress. In the brain, diabetes also impaired mitochondrial function by decreasing both state 4 and state 3 respiration rates and mitochondrial membrane potential while increasing ROS levels and decreasing the GSH (reduced glutathione)/GSSG (oxidized glutathione) ratio. All these alterations induced by T1DM were counteracted by daily administration of AO [175,176]. 

A comprehensive study on Goto–Kakizaki rats, a model for T2DM, explored the long-term effects of AO supplementation over 12 months. The investigations revealed that diabetic rats displayed persistent hyperglycemia and increased markers of oxidative stress in kidney mitochondria, with ROS levels in these rats being 1.6 times higher by the third month and peaking at 90% higher by the twelfth. In addition, even healthy rats exhibited an escalation in ROS levels by the 6-month mark, underscoring the impact of aging. However, AO consumption counteracted these effects, reducing ROS levels by up to 75% at six months and significantly ameliorating hyperglycemia. Concurrently, the aging process and diabetes have been shown to deplete glutathione concentrations, yet AO supplementation has been demonstrated to fully restore glutathione levels at both three and six months, maintaining a level 2.6 times higher than in diabetic controls at 12 months. This finding suggests that AO possesses significant potential as a dietary antioxidant in mitigating oxidative stress, a key factor in the development of aging-related diseases. While AO did not restore glucose levels to normal levels, its partial hypoglycemic effect could have a beneficial effect on cardiovascular health, as each 25 mg/dL increase in glucose was associated with a 15% increase in cardiovascular risk [177]. Consequently, the reduction in blood glucose levels induced by AO has the potential to decrease cardiovascular risk in aging organisms and in the management of diabetes-related complications associated with aging. The reduction in glucose levels by AO agrees with another study where dietary supplementation with AO led to a substantial reduction in insulin resistance in rats consuming fructose [178]. Accordingly, recent studies by our research group demonstrated that an unsaponifiable fraction of AO decreased insulin resistance in rats consuming a high-fat, high-fructose diet, suggesting that these effects may be independent of fatty acids from AO [107]. 

Another investigation into the impact of AO on diabetic nephropathy in Goto–Kakizaki rats produced substantial findings concerning renal health. The research highlighted that T2D rats exhibited persistent renal injury, characterized by increased proteinuria and low levels of serum adiponectin, indicative of mitochondrial dysfunction and oxidative stress. Remarkably, the administration of AO resulted in a substantial amelioration of these deleterious effects. AO supplementation not only increased adiponectin levels, which were initially low in diabetic rats, but this increase was associated with a reduction in proteinuria and a mitigation of renal damage. These observations suggest that AO may enhance renal function by potentially improving mitochondrial function, elevating adiponectin levels, and reducing oxidative stress, offering a promising dietary strategy for managing diabetic kidney disease [179].

### 6.3. AO and Its Effects Against NAFLD 

AO supplementation significantly improved NAFLD in rats fed a high-fat, high-fructose diet (HFHFr). AO enhanced mitochondrial respiration and the activity of the electron transport chain (ETC) complexes and reduced the levels of ROS and lipid peroxidation. AO attenuated hepatic steatosis, hypertrophy, and inflammation, as evidenced by histological analysis, which showed a reduction in microvesicular steatosis from 60% to less than 20% of hepatocytes, macrovesicular steatosis from 80% to 15%, and a reduction in hepatocyte ballooning from 70% to 60% in the HFHFr group. Furthermore, AO not only counteracted the deleterious effects of the HFHFr but also reversed hyperglycemia, dyslipidemia, and mitochondrial dysfunction when included in the diet, demonstrating its potential to not only manage but potentially reverse NAFLD. In addition, AO reduced the expression of pro-inflammatory markers such as TNF-α and IL-6 in the liver, suggesting its broader therapeutic potential in the management of inflammation associated with aging and chronic disease [180,181].

### 6.4. AO in Hypertensive Models

Hypertension is a condition highly related to aging [182]. AO has shown significant potential to improve vascular and mitochondrial function, reduce oxidative stress, and protect against renal damage in hypertensive rat models. There is a recognized association between a 10 mmHg reduction in diastolic blood pressure and reduced cardiovascular risk. In these studies, AO was shown to reduce both systolic and diastolic blood pressure by 15.5% and 21.2%, respectively, which directly correlates with a reduction in this risk. AO also enhanced renal endothelium-dependent vasodilation, which has a beneficial effect on glomerular function and structure. This was linked to a decrease in oxidative stress, with AO improving mitochondrial function, reducing ROS production, and improving vascular health, thereby contributing to the improvement in renal function. At the mitochondrial level, AO prevented the loss of membrane potential by 83.7% and reduced ROS by 51% when mitochondria were energized with complex I substrates. It also restored the redox state of glutathione, reducing the level of oxidized glutathione by 48%. These effects are mainly attributed to the high levels of oleic acid and a variety of antioxidants in AO, such as carotenoids, which can attenuate the conversion of nitric oxide to peroxynitrite, thereby reducing oxidative stress and potentially alleviating endothelial damage. Interestingly, the effects of AO on mitochondrial function and oxidative stress mimic those of losartan, an angiotensin II antagonist, suggesting that AO may exert its benefits by counteracting the actions of angiotensin II at the mitochondrial level [183].

### 6.5. Age-Related Hearing Loss 

A study has investigated the effects of AO in the prevention and treatment of sensorineural hearing loss in vitro and in vivo. An enhanced AO extract, called DKB122, was found to have the ability to restore damaged auditory hair cells in zebrafish and improve hearing function in mice with noise-induced hearing loss. The study also demonstrated that DKB122 exerts its effects by modulating the expression of genes associated with resistance to oxidative stress, cytokine production, and amino acid biosynthesis, thereby suggesting a protective mechanism against cell damage in the inner ear. These findings position AO as a potential therapeutic agent for age-related hearing loss, thus adding a new dimension to its beneficial profile in the context of age-related diseases [184].

Furthermore, additional research has investigated the components of AO that are responsible for this protective effect. A study identified 20 compounds in AO, including two new fatty acid derivatives (namely, (2R,4R,6Z)-1,2,4-trihydroxynonadec-6-ene and (2R,4R)-1,2,4-trihydroxyheptadecadi-14,16-ene), which showed significant protection against neomycin-induced hair cell damage in a zebrafish model. This finding lends further credence to the therapeutic potential of AO derivatives in mitigating hearing loss, suggesting that both the oil itself and its isolated components could be beneficial in managing or preventing age-related hearing impairments [185].

### 6.6. The Potential of AO in Mitigating Neurodegeneration 

AO has demonstrated the ability to attenuate neurodegeneration induced by atrazine, a neurotoxic herbicide, by reducing glutamate levels associated with excitotoxicity. AO also modulates acetylcholinesterase activity, which is crucial for brain function in aging. In the hippocampus, AO preserves neurons, reduces inflammation, and improves cognitive performance. These findings suggest that AO might help mitigate age-related cognitive decline by potentially reducing oxidative stress and inflammation, thus supporting brain health in aging or at-risk individuals. However, these benefits are suggested based on animal models, and further research is required to translate these effects to human aging [186].

### 6.7. The Potential of AO in Accelerating Wound Healing in Skin

Aging leads to reduced collagen synthesis, slowing healing, weakening scar tissue, and increasing infection risk due to lower skin tensile strength. Preclinical studies have shown that AO significantly enhanced healing by increasing collagen density, reducing inflammation, and accelerating wound contraction and re-epithelialization. AO also improved tensile strength, suggesting better scar tissue formation, making it a valuable option for managing skin wounds in the elderly, enhancing healing speed and scar quality [187].

Overall, preclinical evidence demonstrates the broad therapeutic potential of AO in aging-related diseases. This potential is highlighted by the ability of AO to reduce oxidative stress; enhance mitochondrial function; and manage conditions such as diabetes, NAFLD, hypertension, hearing loss, impaired wound healing, and neurodegeneration.

**Figure 2:**
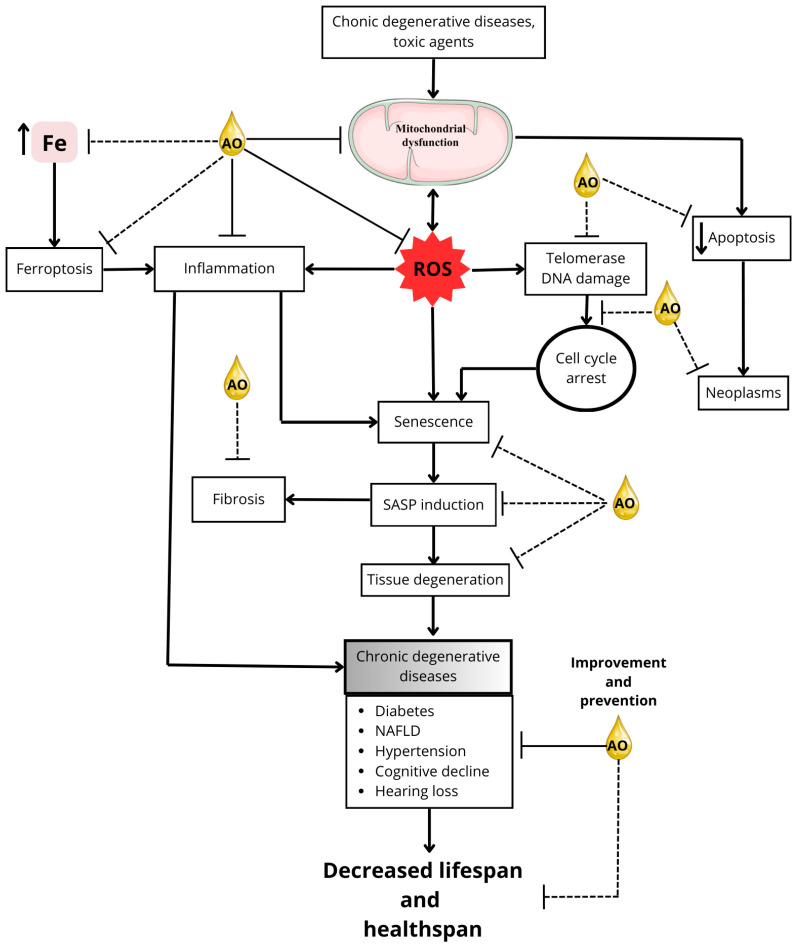
Mitigating aging and age-related diseases: potential AO targets. This figure illustrates the potential targets by which AO compounds might prevent or delay aging processes, thereby promoting an extension of healthspan and possibly lifespan. Solid lines in the figure represent mechanisms for which research supports the action of AO, whereas dotted lines indicate mechanisms or interactions that are not yet fully elucidated. The direction of the arrows shows the direction of the phenomenon being indicated; an upward pointing arrow, for example, indicates an increase, while a downward pointing arrow indicates a decrease. The double arrow indicates that the phenomenon may be either way.

Figure 2 demonstrates the potential of AO in mitigating the effects of aging and age-related diseases.

## 7. Conclusions 

Based on the information in this review, it is feasible to propose that AO may have an anti-aging effect due to the extension of longevity observed with some of the antioxidants and fatty acids present in AO in aging models (Table 2). In addition, regular consumption of AO could be a coadjuvant to a healthy lifestyle to prolong healthspan, since some of its components can improve telomerase activity [134], antioxidant systems [85,86,188], autophagy [189], hepatic detoxification of xenobiotics [115], ferroptosis [73,91,93], and inflammation [54,84,102] (Figure 1 and Table 1). In support of this hypothesis, individual components of AO have been observed to have therapeutic potential in pathological conditions associated with aging, such as neurodegenerative diseases [125], loss of cognitive function [124], rheumatoid arthritis [148], cancer [165,166], atherosclerosis [129,156], diabetes [107,177,178], hypertension [190], and myocardial remodeling [191].

The hypothesis that AO could prolong healthspan by ameliorating chronic degenerative diseases carries more weight considering that direct effects of AO consumption have been described to lower blood pressure in hypertensive rats [183,192]; improve blood glucose and adiponectin levels in lean diabetic rats [179,193] and in diabetic rats on a high-fat, high-fructose diet [181]; reduce insulin resistance [107,194,195] and inflammation levels in various organs in models of obesity and NAFLD [181,195]; improve cognitive performance [195]; and protect against hearing loss [184,185]. In addition, its protective effects against mitochondrial dysfunction and oxidative stress have been implicated in the prevention of diabetic and hypertensive nephropathy and NAFLD [179,181,192].

There is still a lack of information on the effects of AO on other processes closely related to aging (Figure 1), such as SASP secretion by senescent cells; fibrogenesis; genetic, epigenetic, and telomeric damage leading to cell senescence; as well as its effect on cellular quality control processes that are impaired in aging, such as autophagy, mitophagy, and proteostasis. The effects of AO on cell death processes that may be dysregulated in aging, such as necrosis, ferroptosis, and apoptosis, are also unknown. In addition, there is no data on the effects of AO on calcium homeostasis, which is consistent with the lack of data on the effects of AO on cardiac function [54]. Although the effects of AO on the increase in intracellular free iron in aging are also unknown, some data suggest that AO may decrease susceptibility to ferroptosis, since it has been shown that AO treatment decreases lipid peroxidation induced by excess iron and increases GSH levels [175,193]. On the other hand, the lower mitochondrial production of ROS [176,181,183,192,193,195] and decreased levels of lipid peroxidation [175,181,193] and inflammatory markers by AO [181,194] suggest that its consumption could attenuate or delay the induction of SASP, thereby decreasing senescence. 

When considering the consumption of AO and its components, safety is an essential factor. While AO is generally considered safe for consumption, certain components like PAc have been associated with pro-inflammatory and cytotoxic effects [75,76]. Therefore, it is advisable to consume AO in adherence to recommended dietary guidelines. It is noteworthy that AO shares a similar fatty acid profile with olive oil, which is recognized as safe and beneficial for cardiovascular health when consumed in amounts up to four tablespoons (50 g/day) [196,197]. 

The AO market is expanding rapidly, driven by increased demand from the cosmetics and food industries as well as its recognition as a functional food with documented health benefits. While the use of avocados for oil production is increasing, it is anticipated that global production of avocado fruits may not be sufficient to meet the growing demand for AO [54].

A brief comparison of the effects of AO with those of other fruit oils on health outcomes is pertinent. Olive oil, which shares desirable features like a high MUFA content with AO, has been linked to reducing hypertension, managing diabetes, improving cognitive function, and potentially preventing cancer [166,198,199,200]. Among fruits, avocado and olive oil are the most commonly used oils due to their oleaginous nature. Furthermore, almond oil is another oil that has been shown to activate NRF2 and improve antioxidant defenses in diabetic rats [201]. In epidemiological and clinical studies, almond oil consumption resulted in a substantial decrease in plasma triglycerides, total cholesterol, and LDL cholesterol together with an increase in HDL cholesterol [202]. Conversely, coconut oil, characterized by its high saturated fat content (approximately 92%) [203], has been linked to an increase in LDL cholesterol and is regarded as detrimental to cardiovascular health. Consequently, it is recommended that coconut oil be replaced with oils that are rich in polyunsaturated fats in order to promote health benefits [204]. This highlights the significant anti-aging properties of AO from other fruit oils.

In conclusion, the beneficial effects of AO and its individual components on various processes involved in the progression of aging, its protective effect against the development of diseases and physiological alterations associated with aging, and its low toxicity [205,206] make it a promising nutritional agent for prolonging healthspan during aging and/or an interesting source of novel molecules with anti-aging properties and pharmaceutical potential.

## Figures and Tables

**Table 1:** AO compounds for anti-aging and age-related diseases.

Compounds	Source of Compound	Model Study	Outcome/Mechanisms Involved	Age-Related Disease	References
Fatty acids					
OAc	From diet	Elderly population cohort study	Better cognitive function according to Montreal Cognitive Assessment and improvement in logical memory function	Cognitive decline	[124]
	Pure compound > 99% (Sigma-Aldrich, St. Louis, MO, USA)	Neuron-like cells model of neurotoxicity induced by Aβ fragment 25–35	Neuroprotective effect and attenuation of intracellular ROS levels and downregulation of pro-apoptotic activated caspase-3, mitigating apoptotic morphological alterations as well as improvement of neuronal damage associated with COX-2 and iNOS downregulation through activation of NF-κB.	AD	[68]
	Pure compound (Nu-checkprep)	Fibroblast-like cell line COS-7 and C57BL6/J transgenic mice	Aβ secretion levels were reduced in cells, while in mice, an increased Aβ40/Aβ42 ratio was observed, accompanied by reduced levels of beta-site APP cleaving enzyme and presenilin, along with a decrease in amyloid plaques in the brain, reducing Alzheimer’s disease neuropathology.	AD	[125]
POAc and OAc	Pure compound (Sigma-Aldrich)	Prediabetic model of hereditary hypertriglyceridemic rats	POAc enhanced insulin sensitivity in adipocytes, facilitating improved glucose uptake and metabolism, exhibiting anti-inflammatory properties by reducing the expression of pro-inflammatory cytokines in adipocyte cells. Regarding OAc, improved insulin sensitivity.	Prediabetes	[126]
Linoleic acid	Pure compound (Tokyo Chemical Industry, Tokyo, Japan)	Sprague-Dawley rats and Caco-2 cells	Reduction in postprandial blood glucose through the reduction of GLP-1 and slowing gastric emptying.	Type 1 diabetes	[127]
Linolenic acid	Pure compound	*C. elegans* muscles	50 μg/mL improved sarcopenia by repairing mitochondrial function by promoting mitophagy and fighting oxidative stress.	Sarcopenia	[79]
α-linolenic acid	Pure compound (Sigma-Aldrich)	Human astrocyte cells SH-SY5Y with induction of cell death with Aβ_1-42_	Protection against mitochondrial dysfunction through stimulating release of insulin and IGF-1 from astrocytes.	AD	[81]
Phytosterols					
β-sitosterol	Pure compound (Shanghai Yuanye Bio-Technology Co., Shanghai, China)	Zebrafish larvae model of inflammation and oxidative stress induced with CuSO_4_	40 mg/kg of β-sitosterol treatment reduced the level of interleukin ROS markers while increasing antioxidant enzyme expression levels and may induce an anti-nociceptive effect via inhibiting IL-6, oxidative stress, COX, and NO.	Inflammatory diseases	[86]
β-sitosterol	Pure compound (Sigma-Aldrich)	Macrophages RAW 264.7	3 h of β-sitosterol pretreatment showed antioxidant and anti-inflammatory activities through GPx over expression.	Inflammatory diseases	[85]
β-sitosterol	Pure compound (by BASF China, Nanjing, China)	Wistar rats induced by a high-fat diet	β-sitosterol reduced endoplasmic reticulum stress by preventing the overexpression of IRE-1α, sXBP1, and CHOP.	NAFLD	[128]
β-sitosterol	Pure compound (Traditional Chinese Medicine Systems)	Apolipoprotein E knockout mice and vascular smooth muscle cells (VSMCs)	β-sitosterol alleviates atherosclerosis by regulating catalase activity, leading to reduced lipid deposition and phenotypic transformation of VSMCs and suppression of the PI3K/AKT/mTOR signaling pathway.	Atherosclerosis	[129]
β-sitosterol	*Indigofera zollingeriana* plant extract	HepG2 and Huh7 cells	Anticancer effect through activating the caspase-3 and -9 signaling pathways associated with apoptosis cell death.	Hepatic cancer	[130]
Tocopherols					
α- and γ-tocopherols	Pure compounds (Sigma-Aldrich)	Murine microglial BV-2 cells	α- and γ-tocopherols improved 7-KC-induced loss of transmembrane potential, which is associated mitochondrial with cell death, and they prevented the decrease in Abcd3 protein levels, which allows for the measurement of peroxisomal mass.	Neurotoxicity	[90]
α- and γ- tocopherols	Tocopherols in the brain comes from diet	113 deceased participants from the Memory and Aging Project in Rush	Higher α- and γ-tocopherol levels were associated with lower total and activated microglia density in cortical but not in subcortical brain regions, suggesting that the relation between tocopherols and AD might be partly explained by the alleviating effects of tocopherols on microglia activation.	Cognitive decline from dementia and AD	[131]
α-tocopherol	Gelatin capsules of α-tocopherol with pioglitazone	Trial on human adults with NAFLD without diabetes and cotreatment with pioglitazone	Improvement of steatosis scores and lobular inflammation in histologic features on NAFLD.	NAFLD	[132]
Carotenoids					
Lutein	FloraGLO Lutein	Randomized controlled clinical research	Supplementation of 20 mg/day for 40 days improved attention and memory and showed cognitive benefits, such as protection from neuronal damage.	Multiple sclerosis and age-related neurodegenerative diseases	[133]
Lutein	Pure compound (Sigma-Aldrich)	Mesenchymal stem cells	β-carotene regulated the KAT7-P15 signaling axis, modulating the expression or activity of P15, enhancing inflammation and oxidative stress.	Aging	[102]
β-carotene	Plasma levels of β-carotene from dietary sources	68 older subjects, 37 with Alzheimer’s and 31 age-matched healthy controls	β-carotene plasma level, LTL, and peripheral telomerase activity were measured and showed improvement.	Alzheimer’s disease	[134]
Chlorophylls					
Pheophytin *a* and hydroxy pheophytin a	*Thunbergia laurifolia* leaves	HepG2 cell	Potent NQO-1 inducers resulting in liver detoxification.	Hepatic cancer	[115]
Xanthones	Stem bark of *Garcinia smeathmannii*	RAW 264.7 macrophages	Ananixantona showed inhibition of NO production with inhibitory effects on LOX activity in activated macrophages.	Inflammatory disease	[135]
Phenolic compounds	Pure compounds (Sigma-Aldrich)	*C. elegans*	Phenolic acids (vanillic and gallic acid) significantly increased the lifespan, improved mitochondrial function, and activates longevity pathways via hormesis, possibly through sirtuin pathway engagement.	Aging	[121]

Aβ: beta-amyloid peptide; ROS: reactive oxygen species; COX-2: Cyclooxygenase-2; AD: Alzheimer’s Disease; iNOS: inducible nitric oxide synthase; NF-κB: nuclear factor kappa B; APP: amyloid precursor protein; POAc: palmitoleic acid; GLP-1: glucagon-like peptide-1; IGF-1: insulin-like growth factor-1; GPx: glutathione peroxidase; IRE-1α: inositol-requiring enzyme-1; sXBP1: X-box binding protein 1; CHOP: C/EBP homologous protein; VSMCs: vascular smooth muscle cells; 7-KC: 7-Ketocholesterol; Abcd3: ATP binding cassette subfamily D member 3; NAFLD: non-alcoholic fatty liver disease; KAT7-P15: lysine acetyltransferase 7; LTL: leucocyte telomere length; NOQ-1: NAD (P)H quinone oxidoreductase 1; NO: nitric oxide; LOX: lysyl oxidase.

**Table 2:** Effect of bioactive compounds on aging and lifespan models.

Model	Compound and Source	Outcomes/Mechanism	Reference
*C. elegans*	OAc pure compound from Sigma-Aldrich	Promotes longevity by activating SKN-1A transcription factor, enhancing lipid homeostasis, and reducing fat accumulation.	[136]
*C. elegans* and *C. remanei*	OAc pure compound in ethanol solution	Extends lifespan by activating FAT-7, a desaturase enzyme which promotes fat storage and counteracts a post-mating life reduction.	[137]
*C. elegans*	n-6 fatty acid pure compounds from Cayman chemical^®^ in ethanol solution	Extend lifespan through the activation of autophagy.	[138]
*C. elegans*	PAc from sea cucumber *Holothuria scabra*	Extends lifespan and increases stress resistance via DAF-16/FOXO and SKN-1/NRF2 pathways.	[77]
*C. elegans*	α-linolenic acid from Cayman chemical^®^	Extends lifespan via mechanisms involving the NHR-49/PPARα and SKN-1/Nrf2 transcription factors.	[140]
Mesenchymal stem cells and male C57 mice	β-carotene pure compound from Sigma-Aldrich	Anti-aging effect through regulating the KAT7-P15 signaling axis, inflammation, and oxidative stress process.	[102]
*C. elegans*	β-carotene, canthazanthin, and astaxanthin extracted from microalga *Haematococcus lacustris*	Lifespan extension by 1.3-fold via upregulation of SOD expression.	[141]
*D. melanogaster*	Lutein pure compound from the Chenguang Biotech Group	Lifespan-prolonging activity of lutein was partially attributed to up-regulation of antioxidant enzymes.	[142]
*C. elegans*	Chlorophyll from spinach	Enhances the lifespan up to 25% via activation of DAF-16/FOXO-dependent pathway.	[143]
*D. melanogaster*	Phytosterols from mountain-cultivated ginseng	Activation of steroid signaling pathways, mimicking effects of steroid hormones and enhancing longevity.	[145]
*C. elegans*	Phenolic acids (vanillic and gallic acid) pure compounds from Sigma-Aldrich	Activate longevity pathways via hormesis, possibly through sirtuin pathway engagement.	[121]
*D. melanogaster*	β-sitosterol, flavonoids, and polyphenols from *Ludwigia octovalvis*	Extending lifespan, likely through activating AMPK in body fat.	[147]

## Data Availability

No new data were created or analyzed in this study. Data sharing is not applicable to this article.
